# Evaluation of prediagnosis emergency department presentations in patients with active tuberculosis: the role of chest radiography, risk factors and symptoms

**DOI:** 10.1136/bmjresp-2016-000154

**Published:** 2017-01-17

**Authors:** S C Appleton, D W Connell, A Singanayagam, P Bradley, D Pan, F Sanderson, B Cleaver, A Rahman, O M Kon

**Affiliations:** 1 Tuberculosis Service, Imperial College Healthcare NHS Trust, London, UK; 2 Department of Emergency Medicine, Imperial College Healthcare NHS Trust, London, UK

**Keywords:** Infection Control, Tuberculosis

## Abstract

**Introduction:**

London has a high rate of tuberculosis (TB) with 2572 cases reported in 2014. Cases are more common in non-UK born, alcohol-dependent or homeless patients. The emergency department (ED) presents an opportunity for the diagnosis of TB in these patient groups. This is the first study describing the clinico-radiological characteristics of such attendances in two urban UK hospitals for pulmonary TB (PTB) and extrapulmonary TB (EPTB).

**Methods:**

We conducted a retrospective cohort study using the London TB Register (LTBR) and hospital records to identify patients who presented to two London ED's in the 6 months prior to their ultimate TB diagnosis 2011–2012.

**Results:**

397 TB cases were identified. 39% (154/397) had presented to the ED in the 6 months prior to diagnosis. In the study population, the presence of cough, weight loss, fever and night sweats only had prevalence rates of 40%, 34%, 34% and 21%, respectively. Chest radiography was performed in 76% (117/154) of patients. For cases where a new diagnosis of TB was suspected, 73% (41/56) had an abnormal radiograph, compared with 36% (35/98) of patients where it was not. There was an abnormality on a chest radiograph in 73% (55/75) of PTB cases and also in 40% (21/52) of EPTB cases where a film was requested.

**Conclusions:**

A large proportion of patients with TB present to ED. A diagnosis was more likely in the presence of an abnormal radiograph, suggesting opportunities for earlier diagnosis if risk factors, symptoms and chest radiograph findings are combined.

**Key messages**

- 39% of tuberculosis cases present to A+E in the preceding 6 months.
- Symptoms alone are insuffiently sensitive to rule out TB in an acute setting.
- Plain chest radiography in combination with risk factors and symptoms offers the best diagnostic yield.

## Introduction

The WHO estimated that 9.6 million people developed tuberculosis (TB) in 2014.^1^ In England, the incidence of TB is persistently high. In 2014, 6520 cases of TB were reported.^2^ For the same year in the USA, which has a population nearly five times that of the UK's, 9421 cases of TB were reported^3^. Countries similar to the UK have made steady but intensive efforts to control TB and have seen a reduction in the number of new cases.^4^

*Mycobacterium tuberculosis* infection may have a prolonged subclinical phase during which the diagnosis and isolation of infectious cases can be difficult. In addition, current diagnostic methods have either a low specificity (eg, chest radiographs/Interferon Gamma Release Assays (IGRA)) or there is a long delay until results are available, especially when sputum smear results are negative and microbiological culture can take several weeks. These factors all contribute to making screening programmes for active TB more challenging to implement.^5^ Therefore, identification of factors that will lead to the consideration of a diagnosis of TB may prompt a more rapid initiation of investigations and sampling to allow for TB control measures.

In the UK, the majority of new cases are concentrated in densely populated, urban areas. In 2014, London saw 2572 new cases of TB, nearly 40% of the national total, a rate of 30.1 per 100 000 which is nearly three times that of the UK average (12.0 per 100 000). The majority of these cases were in patients who were born outside the UK; the incidence was also higher in patients who were homeless, drug and alcohol misusers or immunosuppressed.^2^ The UK health authorities already recognise the difficulty in identifying and treating these patient groups and have released specific guidance to achieve early identification and successful treatment.^6^ The emergency department (ED) is an important facet of healthcare usage for these patient groups who do not regularly attend other healthcare services such as general practitioners (GPs) or may not be registered.^7^ ^8^ ^9^

There have been a small number of US-based studies looking at ED presentations of pulmonary TB (PTB), finding a high frequency of these patients born outside the USA, and with pulmonary symptoms and an abnormal chest radiograph increasing the likelihood of a TB diagnosis.^10^ ^11^ In the UK, although one study has investigated TB symptoms and blood results in inpatients,^12^ and two short reports have examined cases at ED presentation,^13^ ^14^ we have made a specific analysis of the usefulness of risk factors, symptoms and chest radiographs in the diagnosis of PTB and extrapulmonary TB (EPTB) in the ED.

The aim of this study was to evaluate prediagnosis ED attendances in patients with a final diagnosis of confirmed active TB infection, to identify factors that are associated with delayed diagnosis, and investigate strategies to help identify such cases.

## Methods

### Study population

We performed a retrospective cohort analysis of patients that were diagnosed with active TB at two large central London hospitals between 1 January 2011 and 31 December 2012. ED discharge records were then used to identify patients with TB who had visited the ED in the previous 6 months. No age restriction was applied. Patients were only discounted from the study if there were no records available; this occurred in two cases. The study was approved as a service evaluation and ethics committee approval was not required.

### Data collection

The London TB Register (LTBR) is a database recording new TB cases and demographic, comorbidities, disease and outcome data; this along with ED notes, blood results and imaging databases were used in our data collection. We recorded the presence of a number of factors of interest, including demographic factors such as age, gender, country of birth, ethnicity, English speaking and number of years since entry into the UK. We also included known TB risk factors (eg, previous TB, TB contacts, alcohol misuse, homelessness and so on) as well as baseline physiological observations taken on presentation to ED and the presence of TB-related symptoms (cough, subjective fevers, night sweats, weight loss, sputum production, dyspnoea, haemoptysis and chest pain) and other symptoms. Any abnormal clinical findings related to TB on chest examination (crepitations, reduced air entry, dull percussion note or reduced expansion), lymphadenopathy or tenderness during an abdominal examination, were noted. We recorded whether the ED notes commented on the chest radiograph, the findings of the chest radiograph and any other imaging performed and the outcome of the encounter.

### Radiological evaluation

The chest radiograph reports were reviewed. Any reports that referenced possible TB-related changes, for example, cavitation, infiltrates, effusion, lymphadenopathy or pleural thickening or if the report raised the possibility of TB as the cause were recorded as ‘abnormal’. If no reference to TB-related changes was made or the report found no abnormalities, it was recorded as ‘normal’.

### Statistical methods

All data are presented as n (%) unless otherwise stated. The χ^2^ test or Fisher's exact test was used to compare categorical variables. The Mann-Whitney U test was used to compare continuous variables. Multivariable analysis was used to evaluate the association of variables recorded on presentation with suspicion of TB diagnosis in ED. The variables were included in the regression model are shown in online supplementary table S1. The threshold for statistical significance was p<0.05.

10.1136/bmjresp-2016-000154.supp1 supplementary table

## Results

### Study cohort

There were 397 patients included in the study, out of these 397 cases, 156 (39%) presented to the ED in the 6 months prior to their diagnosis. Two cases were excluded due to inadequate records being available. Baseline demographics of the study population are shown in table 1.

**Table 1:** Baseline demographics of study cohort

Demographics/patient factors	Frequency (%)
Population size	397
Male	231 (58%)
Median age (IQR)	35 (25)
Pulmonary TB	166 (42%)
Non-pulmonary TB	231 (58%)
Indian subcontinent	117 (29%)
Black African	111 (28%)
White	74 (19%)
Black Caribbean	22 (6%)
Arabic	26 (7%)
Other ethnicity	47 (12%)
Born outside UK	309 (78%)
Previous BCG vaccination	223 (56%)
Previous TB infection	16 (4%)
Unemployed	67 (17%)
Homelessness	29 (7%)
Drug misuse	22 (6%)
History of imprisonment	17 (4%)
Not registered with GP	79 (20%)

GP, general practitioner; TB, tuberculosis.

### Comparison of patients with TB who presented to ED prior to diagnosis with those who did not

There were 154 (39%) of the study population who presented to ED in the 6-month period prior to their diagnosis of TB. Table 2 shows comparison of demographic and comorbid variables between patients who attended ED versus those who did not.

**Table 2:** Baseline demographics and patients factors in subgroup who attended ED versus group who did not

	Attended ED (%)	Did not attend ED (%)	χ^2^ test (p value)
Demographics			
Population size	154	243	–
Male	100 (65%)	131 (54%)	0.030
Age (IQR)	34 (23)	35 (22)	0.408
Pulmonary TB	75 (49%)	91 (37%)	0.045
Non-pulmonary TB	79 (51%)	152 (63%)	
Ethnicity			
Indian subcontinent	40 (26%)	77 (32%)	0.224
Black African	44 (29%)	67 (28%)	0.829
White	31 (20%)	43 (18%)	0.544
Black Caribbean	12 (8%)	10 (4%)	0.119
Arabic	13 (8%)	13 (5%)	0.225
Other ethnicity	14 (9%)	33 (14%)	0.177
Comorbidities			
Born outside UK	120 (78%)	189 (78%)	0.973
Previous BCG vaccination	89 (58%)	134 (55%)	0.604
Previous TB infection	9 (6%)	7 (3%)	0.143
Unemployed	27 (18%)	40 (16%)	0.781
Homelessness	12 (8%)	17 (7%)	0.766
Drug misuse	11 (7%)	11 (5%)	0.267
Prison history	8 (5%)	9 (4%)	0.475
Not registered with GP	38 (25%)	41 (17%)	0.058

ED, emergency department; GP, general practitioners; TB, tuberculosis.

### Symptomatic presentation and baseline observations in patients who attended ED

We next conducted an evaluation of the symptoms reported by patients who attended ED prior to TB diagnosis. Table 3 shows the most common symptoms reported and also the proportion of patients with baseline abnormalities in routine observations. Thirty-one per cent of cases presenting to the ED had no abnormal observations (48/154).

**Table 3:** Symptoms and baseline observations of patients who attended ED prior to diagnosis of TB

Risk factors	Frequency (% of study population)	Pulmonary TB symptoms	Frequency (% of study population)
Current smoker	27 (18%)	Cough	62 (40%)
Recent travel	19 (12%)	Fever	53 (34%)
Known TB contacts	13 (8%)	Weight loss	53 (34%)
Airways disease	12 (8%)	Night sweats	33 (21%)
Diabetes (type 1 or 2)	12 (8%)	Chest pain	31 (20%)
HIV positivity	6 (4%)	Sputum production	28 (18%)
Hepatitis B or C positivity	3 (2%)	Dyspnoea	28 (18%)
Alcohol misuse	13 (8%)	Haemoptysis	11 (7%)
Immunosuppressive treatment	3 (2%)	Other symptoms	
Observations		Gastrointestinal	30 (19%)
Tachycardia (<100/min)	53 (34%)	CNS/headache	16 (10%)
Fever (>38°C)	30 (19%)	Back/joint pain	13 (8%)
Tachypnoea (>20/min)	20 (13%)	Neck lump	8 (5%)
Pulse oximetry <95%	8 (5%)	Generalised weakness and malaise	8 (5%)
Hypotension (workup SBP<90 mm Hg)	3 (2%)	Other	9 (6%)

CNS, central nervous system; ED, emergency department; SBP, systolic blood pressure; TB, tuberculosis.

Of the PTB cases, the three most prevalent symptoms recorded in the ED clerking were cough (64%), weight loss (49%) and subjective fevers (41%). EPTB cases were far less likely to have classical TB symptoms with the three most prevalent of these being subjective fevers (28%), weight loss (20%) and cough (18%). Fifty-eight per cent (90/154) had any of the three most prevalent symptoms (cough, weight loss and fevers). In patients with PTB the incidence was 72% (54/75), compared with the lower rate for EPTB 46% (36/79).

Seventy-five per cent of the EPTB cases had other symptoms recorded, 38% (30/79) had gastrointestinal symptoms, 20% (16/79) had central nervous system (CNS)/headache symptoms and 16% (13/79) had back or joint pain symptoms.

### Comparison of patients who had TB suspected in ED versus those who did not

TB was included in the differential diagnosis in the ED in 56 of 154 cases (36%). Table 4 shows a comparison between patients who had TB suspected in ED versus those who did not.

**Table 4:** Comparison of patients where TB was suspected in the ED and those where it was not

	TB suspected in ED Frequency (%)	TB not suspected in ED Frequency (%)	χ^2^ test (p value)
Demographics			
Population size	56	98	–
Male	37 (66%)	63 (64%)	0.823
Age (IQR)	30.5 (16.75)	36.5 (30.5)	0.276 (Mann-Whitney)
Pulmonary TB	41 (73%)	34 (35%)	**<0.001**
Non-pulmonary TB	15 (27%)	64 (65%)	
Indian subcontinent	14 (25%)	26 (27%)	0.835
Black African	19 (34%)	25 (26%)	0.266
White	10 (18%)	21 (21%)	0.595
Black Caribbean	6 (11%)	6 (6%)	0.306
Arabic	5 (9%)	8 (8%)	0.870
Other ethnicity	2 (4%)	12 (12%)	0.086
Born outside UK	47 (84%)	73 (74%)	0.174
Previous BCG vaccination	37 (66%)	52 (53%)	0.116
Unemployed	13 (23%)	14 (14%)	0.161
Homelessness	5 (9%)	7 (7%)	0.691
Prison history	2 (4%)	6 (6%)	0.711
Comorbidities			
Current smoker	12 (21%)	15 (15%)	0.336
Recent travel	14 (25%)	5 (5%)	**<0.001**
Previous TB infection	6 (11%)	3 (3%)	0.0734
Known TB contacts	11 (20%)	2 (2%)	**<0.001**
Airways disease	6 (11%)	6 (6%)	0.306
Diabetes (type 1 or 2)	4 (7%)	8 (8%)	1.000
HIV positivity	3 (5%)	3 (3%)	0.6686
Hepatitis B or C positivity	1 (0%)	2 (2%)	1.000
Drug misuse	5 (9%)	6 (6%)	0.515
Alcohol misuse	5 (9%)	8 (8%)	0.870
Immunosuppressive treatment	0 (0%)	3 (3%)	0.5541
Observations			
Tachycardia (<100/min)	22 (39%)	31 (32%)	0.336
Fever (<38°C)	13 (23%)	17 (17%)	0.376
Tachypnoea (>20/min)	9 (16%)	11 (11%)	0.389
Pulse oximetry <95%	3 (5%)	5 (5%)	1.000
Hypotension (SBP<90 mm Hg)	1 (2%)	2 (2%)	1.000
Symptoms			
Cough	41 (73%)	21 (21%)	**<0.001**
Fever	30 (54%)	23 (23%)	**<0.001**
Weight loss	33 (59%)	20 (20%)	**<0.001**
Night sweats	25 (45%)	8 (8%)	**<0.001**
Chest pain	14 (25%)	17 (17%)	0.255
Sputum production	18 (32%)	10 (10%)	**<0.001**
Dyspnoea	14 (25%)	14 (14%)	0.097
Haemoptysis	7 (13%)	4 (4%)	0.0991
Chest radiograph			
Chest radiography performed	52 (93%)	65 (66%)	**<0.001**
Normal radiograph (% of those who had radiography performed)	11 (21%)	30 (46%)	0.138
Abnormal radiograph (% of those who had radiography performed)	41 (79%)	35 (54%)	**<0.001**

Bold indicates significant statistics.

ED, emergency department; SBP, systolic blood pressure; TB, tuberculosis.

This rate of clinical suspicion of TB was significantly higher in patients with PTB than those with EPTB (55% vs 19%; p<0.01).

There was also a significant difference between the median time from ED attendance to starting treatment. In patients where TB was suspected, median was 9.75 days (3–12.75), compared with 71.25 days (13–84.25) in those where it was not listed as a differential diagnosis (Mann-Whitney U test, p<0.001.[Fig BMJRESP2016000154F1]

**Figure 1 BMJRESP2016000154F1:**
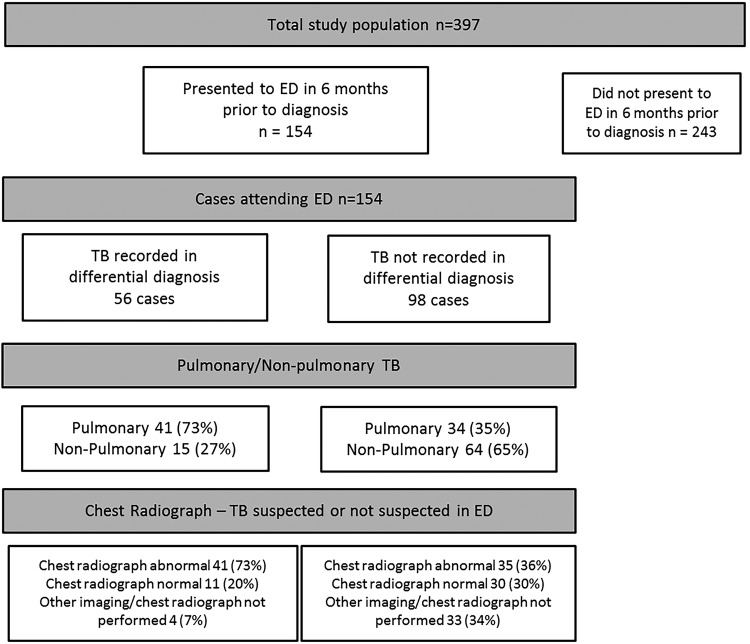
Chest radiograph findings and type of TB in patients presenting to ED. ED, emergency department; TB, tuberculosis.

### Multivariable analysis

On multivariable analysis, the following factors were independently associated with suspicion of TB in ED: cough (OR 6.30 (1.71–23.21), birth outside UK (OR 5.48 (1.29–23.30), known contact with TB infected person (OR 18.63 (2.49–139.27), history of night sweats (16.09 (3.39–76.42) and abnormal chest radiograph (OR 5.90 (1.70–20.53) (see online supplementary table S1).

### Chest radiograph

Of the 154 patients visiting the ED in the 6 months prior to their diagnosis, 24% (37/154) did not have a chest radiograph or other imaging performed on presentation to ED. For PTB cases, 86% (55/64) patients who had a chest radiograph had an abnormality. As would be expected, EPTB had a lower rate of abnormal radiographs, 40% (21/53), but it is notable that there is still a significant proportion with plain chest radiology abnormalities. Of all patients with an abnormal radiograph, many had multiple abnormal findings; the three most common abnormalities seen on radiograph were consolidation in 55% (42/76), effusions in 26% (20/76) and decreased volume or collapse in 22% (17/76). In the patients where TB was not suspected but who had an abnormal chest X-ray (CXR), the most common features were consolidation 46% (16/35), reduced volume 31% (11/35) and effusion 23% (8/35).

Of those 35 patients where the chest radiograph was abnormal and TB was not suspected, 63% (22/35) were men, the average age was 45 years and 71% (25/35) were born outside the UK. Thirty-seven per cent (13/35) of patients had other symptoms present. Fewer patients had any of the three most prevalent symptoms: cough (40%), weight loss (31%) and subjective fevers (29%). Thirty-one per cent (11/35) of these patients reattended the same ED compared with 26% (40/154) overall.

Of patients attending the ED, 69% (107/154) had an abnormal chest radiograph report, presence of cough, subjective fevers or weight loss.

There is no validated probability score in acute TB and we therefore used the features of night sweats and cavitation on chest radiograph as a proxy for cases where TB should have reasonably been suspected. Five per cent (7/154) patients had night sweats and cavitation and all of these patients had TB suspected in the ED. Of the 7% (11/154) patients who had cavitation but no night sweats on chest radiograph, all 11 patients had TB suspected. Eight out of 33 patients who had night sweats but not cavitation did not have TB suspected in the ED.

Forty per cent (21/53) of patients with EPTB who had a radiography performed had an abnormality recorded in the report. Thirty-three per cent (26/79) had no radiography performed.

### Treatment and outcomes

Fifty-four per cent (83/154) of patients were admitted following their accident and emergency (A&E) department attendance and 26% (40/154) of patients reattended A&E following their first presentation. Of those patients who were admitted, 35% (29/83) had TB suspected in the ED and 51% (42/83) had an abnormal CXR compared with 38% (27/71) and 48% (34/71), respectively, for those who were not admitted. The mean interval between presentation to the ED and the initiation of TB treatment was 39 days (range 0–185 days, median 17 days). Thirty-one per cent (48/154) patients were started on TB treatment within a week of their A&E visit. For those patients where TB was considered as part of the differential, the time to starting treatment was dramatically lower, average of 14.4 days compared with 53.6 days.

## Discussion

In our study population, a high proportion of patients, 39%, presented to the ED in the 6 months prior to their diagnosis, demonstrating the importance of this particular location as an opportunity for the detection of patients suffering from this disease. In those suspected to have TB during their attendance, the ED has allowed a timely diagnosis and it is possible that had the individual not accessed healthcare through the ED the case would have remained undiagnosed. In addition, 7% (11/154) had sputum smear performed within 24 hours of attending A&E and these patients may not have had sputum sent in other healthcare settings.

It has also been established that large Western European cities have significantly higher rates of TB infection compared with national rates; London has experienced a continued rise in TB notification rates from 24 per 100 000 in 1990 to 45 per 100 000 in 2011.^15^ ^16^ This high incidence of TB coupled with the high use of emergency care in this urban population emphasises the importance of case detection being initiated promptly by clinicians in the ED.

Our cohort reflected the national picture of TB being more prevalent in patients born outside the UK, homeless, drug and alcohol misusers or immunosuppressed.^2^ This again emphasises the importance of considering TB in any cases presenting with these epidemiological risk factors. A quarter of patients who presented to the ED were not registered with a GP, a higher rate than those not attending the ED. Although the difference was not statistically significant, it highlights that many patients with TB underuse primary care services and are therefore more likely to present to emergency care services.

Apart from male gender, there were no statistically significant differences between the demographics and comorbidities of those who presented to the ED compared with those who did not. Nationally, 50% of ED attenders are men, whereas 65% of the study patients with TB who attended ED were men.^17^ However, the study relied on the accuracy of documentation for these risk factors and comorbidities which will have varied between clinicians.

Pulmonary TB cases represented approximately half of the study population; in this group tachycardia and fever were the most commonly abnormal observations and cough, weight loss and fevers were the most common classical TB symptoms that were present. Almost half of the EPTB patients had no classical TB symptoms and three-quarters presented with other symptoms of which gastrointestinal were the most common. The low incidence rate of classical symptoms, particularly in the EPTB group, makes diagnosis on symptoms alone unfeasible. As would be expected, cases of PTB more frequently displayed chest radiograph abnormalities than EPTB. In keeping with these findings, PTB was more often suspected as a diagnosis in the ED than EPTB (55% vs 19% of cases).

Overall, patients where TB was suspected in the ED had a much greater frequency of chest radiograph, 93% compared with 66%. However, patients where TB was suspected had a greater frequency of all pulmonary symptoms and were therefore more likely to have had a chest radiograph requested. On multivariable analysis, cough, country of birth outside the UK, a TB contact history, a history of night sweats and an abnormal chest radiograph were independently associated with suspicion of TB in ED. The significant proportion of patients where TB was not suspected in the ED but had abnormal radiograph findings (35 patients, 54%) highlights the importance of carefully reviewing all imaging undertaken in the ED and ensuring that any abnormal reports are acted on. Contemporary reporting of imaging while the patient is still in the ED could mean that clinical review can be correlated with radiograph findings. It is important to note that the plain chest radiograph had the best sensitivity when comparing this to symptoms. Importantly, our results indicate that even in cases of EPTB, there is an important role for performing a plain chest radiograph and that this has a broadly similar sensitivity compared with the incidence of classical symptoms. However, the most common chest radiograph abnormalities were consolidation, reduced volume and effusion; these radiographic characteristics can be present for a wide differential of respiratory infections.

This study has a number of strengths and is, to the best of our knowledge, the first study of its kind in the UK to look systematically at radiographs in combination with risk factors and symptoms. We collected and analysed data on a large study population across two sites and it was conducted in a moderately high-incidence urban area. This study also links the initial ED presentation to TB clinic visit events. Previous similar studies have been in lower prevalence areas and in insurance-led healthcare rather than free at the point of use health systems.^10^ ^11^

Other studies in the USA have previously investigated the use of chest radiographs to help TB diagnosis in the ED.^10^ ^11^ These studies found that frequently there is a wide range of symptoms and the chest radiograph helps in assisting the clinician's diagnosis. Our study supports these findings and this is in keeping with recently published national guidelines that encourage a chest radiograph to be performed in all patients with suspected TB (even in cases of EPTB) and to ensure that those with relevant radiographic changes are referred directly to local TB services.^18^ Mobile chest radiographs have previously been shown to have a high sensitivity but in the context of imaging in select high-risk groups.^19^ Our study lends support to interventions such as the ‘Find and Treat’ initiative in London that uses such units to actively find PTB cases.^20^ ^21^

As the study was retrospective, collection of data relied on availability of scanned or electronic records. Importantly, we cannot assume that all patients had active infection with TB 6 months before their diagnosis, or that a diagnosis could have been made on their ED visit. The study only included two EDs and patients may have attended other EDs within the study period and the figure of 39% may underestimate the actual number of visits. The point of contact this group makes with healthcare will increasingly be urgent care centres which were not part of the UK healthcare infrastructure at the time of the study. Also, our study did not have a control group to allow comparisons with the wider population. Ideally, this would be a control group of patients with another respiratory condition, for example, pneumonia which may allow us to look more closely at symptoms and patient factors predicting TB.

The high proportion of patients subsequently diagnosed with TB, that present to the ED prior to their diagnosis, lends support to rapid molecular-based TB diagnostics such as GeneXpert as an adjunct to conventional microscopy, culture and drug sensitivity testing. This would allow timelier testing as part of the workup for patients with suspected TB. It also highlights that patients that are potentially infective are attending a clinical environment where there is a high density of patients and there should be a lower threshold for consideration of isolation especially in higher prevalence, urban areas.

Our study also shows that in cases of EPTB, there was an even greater range of presenting symptoms potentially making the diagnosis even more challenging. Approximately, half of TB patients presenting to the ED have EPTB and although these patients were less likely to have an abnormal chest radiograph (46% vs 86% in PTB), this yield is still important and a chest radiograph should be recommended in any case of potential EPTB. In addition, this study supports the development of a defined diagnostic pathway between ED and TB services when an individual has epidemiological risk factors for TB. These data also support the need for there to be a rapid and direct referral pathway from radiology to TB services should there be any abnormalities indicating potential TB when formally reported.

## Conclusions

This study highlights the importance of the ED as a common key healthcare point of access for TB patients and demonstrates the value of TB departments working across healthcare services as the patient population may access healthcare in a less traditional way. To improve detection and control rates for TB, the diagnosis should be suspected in any patient with a demographic profile or symptoms associated with TB risk and the ED physician should have a low threshold for further investigations for TB. The results of this study suggest that the classical symptoms of TB alone are insufficient in assessment of patients with suspected disease as it frequently presents in a non-specific manner. Our results support the use of chest radiographs in helping alert a potential diagnosis in PTB and EPTB. Importantly, it also raises the difficulties of diagnosing EPTB given its wide varying presentations and highlights that a plain chest radiograph has the highest sensitivity of all the factors normally reviewed in an ED and should be requested in any possible case even when there are no respiratory symptoms.
