# Supplementary material for: Evaluation of prediagnosis emergency department presentations in patients with active tuberculosis: the role of chest radiography, risk factors and symptoms
Source: BMJ Open Respir Res. 2017 Jan 17;4(1):e000154. doi: 10.1136/bmjresp-2016-000154 (PMC5253606; doi:10.1136/bmjresp-2016-000154)
Supplement: supplementary table [file bmjresp-2016-000154supp_table.pdf]

Supplementary Data:

Table 1: Output from multivariate analysis, target variable 'TB suspected in ED'.

Variable	P-value	Odds Ratio	95% Low. Lim.	95% Up. Lim.
Homeless	0.217	3.42	0.49	24.02
Drug misuse	0.122	8.51	0.57	127.79
Previous BCG	0.674	0.79	0.27	2.33
**Cough**	**0.006**	**6.30**	**1.71**	**23.21**
**Born outside UK**	**0.021**	**5.48**	**1.29**	**23.30**
**Known TB contacts**	**0.004**	**18.63**	**2.49**	**139.27**
Recent travel	0.077	3.69	0.87	15.73
Prison history	0.074	0.06	0.00	1.30
Alcohol misuse	0.615	1.66	0.23	12.11
Fever	0.581	0.69	0.18	2.60
**Night sweats**	**0.001**	**16.09**	**3.39**	**76.42**
Weight loss	0.118	2.38	0.80	7.04
Sputum production	0.443	0.55	0.12	2.51
Dyspnoea	0.264	0.46	0.12	1.81
Haemoptysis	0.729	1.43	0.19	10.65
Chest pain	0.163	0.35	0.08	1.52
**Abnormal chest radiograph**	**0.005**	**5.90**	**1.70**	**20.53**
